# Automatic algorithmic driven monitoring of atrioventricular nodal re-entrant tachycardia ablation to improve procedural safety

**DOI:** 10.3389/fcvm.2023.1212837

**Published:** 2023-07-03

**Authors:** Tsz Kin Tam, Angel Lai, Joseph Y. S. Chan, Alex C. K. Au, Chin Pang Chan, Yuet Wong Cheng, Bryan P. Yan

**Affiliations:** ^1^ Division of Cardiology, Department of Medicine and Therapeutics, Prince of Wales Hospital, The Chinese University of Hong Kong, Hong Kong, Hong Kong SAR, China; ^2^ Heart & Vascular Institute, The Chinese University of Hong Kong, Hong Kong, Hong Kong SAR, China; ^3^ Division of Cardiology, Department of Medicine, Queen Elizabeth Hospital, Hong Kong, Hong Kong SAR, China

**Keywords:** AVNRT, slow pathway modification, heart block, radiofrequency ablation, automatic algorithm

## Abstract

**Background:**

During slow pathway modification for atrioventricular nodal reentrant tachycardia, heart block may occur if ablation cannot be stopped in time in response to high risk electrogram features (HREF).

**Objectives:**

To develop an automatic algorithm to monitor HREF and terminate ablation earlier than human reaction.

**Methods:**

Digital electrogram data from 332 ablation runs from February 2020 to June 2022 were included. They were divided into training and validation sets which contained 126 and 206 ablation runs respectively. HREF in training set was measured. Then a program was developed with cutoff values decided from training set to capture all these HREF. Simulation ablation videos were rendered using validation set electrogram data. The videos were played to three independent electrophysiologists who each determined when to stop ablation. Timing of ablation termination, sensitivity, and specificity were compared between human and program.

**Results:**

Reasons for ablation termination in the training set include short AA time, short VV time, AV block and VA block. Cutoffs for the program were set to maximize program sensitivity. Sensitivity and specificity for the program in the validation set were 95.2% and 91.1% respectively, which were comparable to that of human performance at 93.5% and 95.4%. If HREF were recognized by both human and program, ablations were terminated earlier by the program 90.2% of times, by a median of 574 ms (interquartile range 412–807 ms, *p* < 0.001).

**Conclusion:**

Algorithmic-driven monitoring of slow pathway modification can supplement human judgement to improve ablation safety.

## Introduction

Atrioventricular nodal reentrant tachycardia (AVNRT) is the most common cause of supraventricular tachycardia (SVT) (1). Catheter radiofrequency ablation (RFA) is a proven effective treatment for this condition, with a cure rate of over 95% (2–5). Despite the overall safety profile, heart block still occurs in approximately 1%–2.3% of ablations (6–8). Real-time electrograms (EGM) may show signs of an impending permanent AV block, such as prolongation of the AV interval, antegrade AV block, retrograde VA block, or fast junctional rhythm (9, 10). If any of these high-risk features are present, ablation needs to be stopped immediately in hopes that any damage to the AV node is reversible. A typical ablation procedure requires the operator to simultaneously hold the catheter in position, interpret the real-time x-ray or 3-dimensional mapping system, observe the patient's hemodynamics, and monitor more than 10 channels of real-time EGM recordings. Such multitasking is demanding even for an experienced operator. Hence a delay in ablation termination is not uncommon. Such delay can be due to human error or a slower human reaction time. And this is a well-recognized factor that could lead to permanent AV block (6, 9).

Artificial intelligence (AI) has attracted much research interest in assisting arrhythmia diagnoses in recent years. It was used in assisting diagnosis of SVT and paroxysmal AF during sinus rhythm (11–13). It was also promising in predicting ventricular tachyarrhythmia before its onset (14). However, not much research was done utilizing AI during real-time ablation.

An automatic algorithmic-driven program is a simple form of AI. It can potentially recognize high-risk electrogram features (HREF) during ablation, and command ablation termination before human intervention. However, far field electrogram sensing, unexpected noise during ablation, and changes in EGM amplitudes with premature beats all pose potential challenges for such automation. This study investigates the feasibility of designing a program to recognize HREF with reasonable accuracy and stop ablation before human intervention.

## Materials and methods

This investigator-driven study was carried out in a single tertiary referral hospital for electrophysiology in Hong Kong. Consecutive ablation records from February 2020 to June 2022 in this center were reviewed. All slow pathway radiofrequency ablations for typical and atypical AVNRT were included.

### Ablation procedure for AVNRT

The electrophysiology (EP) study and ablation for AVNRT were performed under fluoroscopic guidance and typically did not involve a 3-dimensional mapping system. During EP study, a decapolar catheter was placed at the coronary sinus (CS), and 3 quadripolar catheters were placed at the right atrium (RA), right ventricle (RV), and HIS positions. The RA quadripolar catheter was removed after AVNRT diagnosis was confirmed with EP study. A non-irrigated ablation catheter (Blazer II asymmetric curve 4, Boston Scientific, MA, USA) was inserted via a long sheath. Ablation was performed in temperature-controlled mode, with maximal power set at 60 W, maximal temperature at 60°C and a time limit of 30 s. 14 electrogram channels were stored in the EP recording system (Labsystem Pro, Boston Scientific, MA, USA). These channels include: 3 channels for surface ECG (lead I, II, V1), 2 channels for ablation (ABLd and ABLp), 2 channels for HIS (HISd and HISp), 5 channels for CS (CS1–2, 3–4, 5–6, 7–8, 9–10, with CS1–2 being the distal bipole), and 2 channels for RV (RVAd and RVAp).

### Electrogram analysis

EGM data was exported from the EP recording system as a. txt file. Each file contained approximately 60 s of the procedure (18 s before ablation and 42 s after ablation started). Because each ablation run was typically bounded by a time limit of 30 s, each exported. txt file was expected to contain all electrograms recorded during the ablation. The data contained 14 EGM channels with signal amplitudes from −32768 to +32768 at a sampling rate of 1,000 Hz. These EGM files were divided into two sets chronologically (one year's worth of data from February 2020 to February 2021 for the training set, and the rest for the validation set).

Ablation runs were reviewed by the program designer to determine if electrogram records were adequate for interpretation. Runs were excluded from the training set if there was missing data or contained significant noise in any of the 14 channels before starting ablation. Since proximal CS channels record smaller far field ventricular signals, CS7–8 was chosen as the atrial signal's primary sensing source. The CS9–10 channel may be outside the CS ostium in some cases and therefore was not selected for program input. RVAd was chosen as the primary sensing source for ventricular signals. CS5–6 and Lead II were used as supplementary sensing sources to tackle noise issues during ablation (see results section). Because the final program only required data from 4 channels (CS 7–8, CS 5–6, RVAd, Lead II), a run in the validation set would only be excluded if there was missing data or noise before ablation in any of these 4 channels. Notably, a particular run was not excluded if noise was encountered only after ablation started.

HREF were manually measured in the training set of ablation runs to determine if ablation termination was required. To maximize the sensitivity for HREF, cutoff values were set 10 ms higher or lower than the most extreme value so the program can capture all HREF.

### Program development to stop ablation

A program was developed in Python (Version 3.9) to read atrial and ventricular signals in real-time. Program design details are described in the Results section. Briefly, the program would first establish a sensing threshold for atrial and ventricular electrograms using the data 6 s preceding ablation start. Electrogram data during ablation was fed into the program every millisecond. The program would then determine the timing of atrial and ventricular signals when the amplitude in the corresponding channels exceeded the sensing threshold. It would then continuously monitor for conditions to stop ablation, namely short AA interval, short VV interval, AV block or VA block (see Supplementary Figures S1–S4). The exact time when the program made the command to stop ablation in the validation set would be recorded at a precision down to 1 ms.

### Determination of ablation stop time by human operator

Although the actual ablation stop time was recorded for each case, this recorded time has a delay of up to 500 ms because of an intrinsic connection delay in the EP recording system. Also, operators may often stop ablation if the catheter has moved, in the absence of HREF. Thirdly, our center occasionally involves a second operator responsible for pressing the button to stop ablation upon receiving a cue from the first operator. The reaction time of this second operator may also prolong the delay. Therefore, to ensure that the human arm of this study was not disadvantaged when compared to the program, the following simulation was implemented.

**Figure 1:**
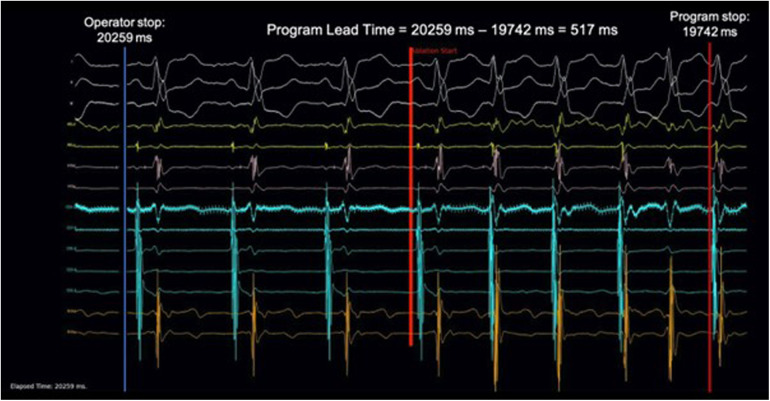
Demonstration of video simulation and calculation of AI lead time. 201 ablation runs in the validation set were rendered into ablation simulation videos at a frame rate of 100 Hz and played to three individual electrophysiologists. A foot pedal was used to control the video. Stepping down on the pedal would begin playing the video and releasing the pedal would stop the video. Upon pedal release, the time at the left lower corner would be recorded as the ablation stop time. This was compared with the ablation stop time determined by the program to calculate the AI Lead Time. A sample of the ablation simulation video can be found in supplementary material.

The exported EGM data was used to plot the 14 EGM channels and rendered into a video with a frame refresh rate of 100 Hz. Efforts were made to resemble real-time ablation as much as possible. The run time was displayed at the bottom left corner of the video. Because the same set of EGM data was used to feed into the final program, the time was synchronized between the video and the program. The video was shown to three independent electrophysiologists, who had no knowledge of program design. All three electrophysiologists were experienced in slow pathway modification procedures. Operator 1 and 2 each performed more than 100 procedures, and operator 3 performed more than 50 procedures. Although this simulation was designed to be intuitive, they each received training with videos generated from the training sets before analyzing the validation sets. They were instructed to step on a foot pedal before ablation started. A red line would be displayed at the start of ablation, and the operators would monitor the electrogram. If they recognized any high-risk features that prompted them to stop the ablation, they would release their foot from the pedal immediately. This action was registered by the computer as a command to pause the video. The ablation stop time at the bottom left corner would then be recorded. With this protocol, the human ablation-stop time could be determined with a precision down to 10 ms. Figure 1 and Supplementary Video S1 illustrate this process.

### Determination of program accuracy

There was no global consensus on cutoff values for HREF. Therefore, the “ground truth” of the presence of HREF was determined by the program designer in the training set. In the validation set, the “ground truth” was a consensus of the three blinded electrophysiologists who had no knowledge of the program design. Upon any discordance in decisions between the human operators, the electrophysiologists were asked to review the videos again. If consensus was still not reached, that ablation run would be excluded from the analysis.

Performance was measured by sensitivity and specificity (Equations 1 and 2). An “AI Lead Time” was calculated in runs where both human and program agreed on the presence of HREF (Equation 3).

$$\begin{array}{rll} & \mathrm{Sensitivity}= & \frac{\mathrm{stop}\;\mathrm{ablation}\;\mathrm{due}\;\mathrm{to}\;\mathrm{HREF}}{\mathrm{stop}\;\mathrm{ablation}\;\mathrm{due}\;\mathrm{to}\;\mathrm{HREF}+\mathrm{continue}\;\mathrm{ablation}\;\mathrm{despite}\;\mathrm{HREF}} \end{array}$$

$$\begin{array}{rl} & \mathrm{Specificity}=\\ & \frac{\mathrm{continue}\;\mathrm{ablation}\;\mathrm{without}\;\mathrm{HREF}}{\mathrm{continue}\;\mathrm{ablation}\;\mathrm{without}\;\mathrm{HREF}+\mathrm{stop}\;\mathrm{ablation}\;\mathrm{without}\;\mathrm{HREF}} \end{array}$$

AILeadTime=timeforhumantostopablation−timeforprogramtostopablation

A post-hoc sensitivity analysis was performed to test consistent program performance across different cutoff inputs Cutoff values were made 10% more stringent or lenient, and the program's sensitivity and specificity of the program was re-evaluated with these new values.

### Statistical analysis

Descriptive statistics were expressed as mean ± SD for data with normal distribution or median (IQR) for skewed distribution. For AI Lead Time evaluation, the null hypothesis assumed no difference between human and AI to identify HREF (AI lead time = 0). One sample *t*-tests and Wilcoxon Signed Rank tests were used. All statistical analyses were performed using SPSS statistical software (IBM SPSS Statistics for Windows, Version 22.0. Armonk, NY: IBM Corp). All analyses were two-tailed and *p*-values of <0.05 were considered statistically significant.

### Research ethics

Research ethics was approved by the local institutional review board (CUHK-NTEC clinical research ethics committee) under CREC No. 2022.505. This study was conducted in accordance with the principles of the Declaration of Helsinki.

## Results

There were 62 slow pathway modification procedures during the study period. Of these procedures, 57 ablations were for typical AVNRT and 5 ablations were for atypical AVNRT. There were a total of 332 ablation runs. The training set and validation sets consisted of 126 and 206 runs respectively.

### Analysis of training set

Of the 126 runs in the training set, 9 ablation runs had at least one missing channel, 5 had channels with uninterpretable signals, and 10 had channels with signal-to-noise ratio less than 1. After these exclusions, 102 ablation runs remained in the training set. Of these runs, 37 contained HREF resulting in ablation termination. The HREF included AV block in 6 runs, VA block in 28 runs, and fast junctional beats (without AV or VA block) in 3 runs. The 6 AV block runs were analyzed as prolongation of AV time (4 runs) or having atrial counter—ventricular counter >= 2 (2 runs). 28 VA block runs were analyzed as prolongation of VA time (20 runs) or ventricular counter—atrial counter >= 2 (8 runs). 3 fast junctional beats without AV or VA blocks runs were analyzed as short AA interval (2 runs) or short VV interval (1 run). To maximize program sensitivity, cutoff values were set 10 ms higher or lower than the most extreme value to ensure all HREF can be captured: A to A interval <240 ms for short AA, A to V interval >250 ms for AV block, V to V interval <220 ms for short VV, V to A interval >200 ms for VA block. The features of the high-risk electrograms and corresponding cutoff values are summarized in Table 1.

**Table 1:** Breakdown of high-risk electrogram features in training set.

HREF	Features and ranges of measurements	Count	Cut-off value for program so that all HREF can be captured
AV block	AV interval 260 ms–520 ms	4	>250 ms
	A-V count >= 2	2	>= 2
VA block	VA interval 210 ms–460 ms	20	>200 ms
** **	V-A count >= 2	8	>= 2
Short AA	AA interval 210 ms–230 ms	2	<240 ms
Short VV	VV interval 210 ms	1	<220 ms
Total		37	

### Program design

The design borrowed the concept of pacemaker sensing to simplify the program and allow for an instantaneous reaction to HREF. The program would primarily use timing from atrial and ventricular signals to determine whether stopping ablation was necessary.

**Figure 2:**
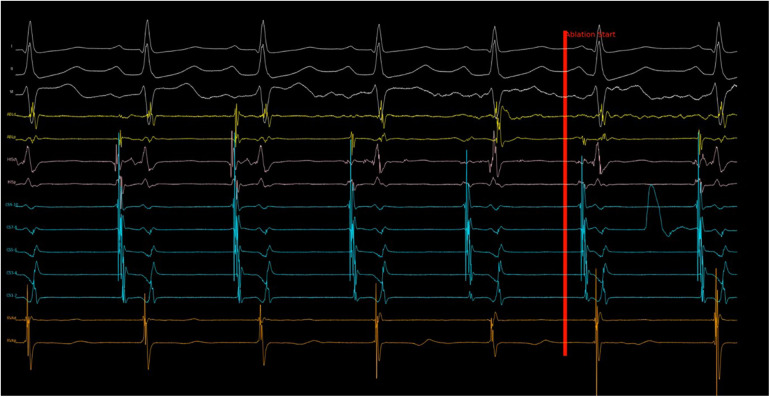
Program response to noise in sensing channel. Shortly after ablation started, noise with large amplitude was recorded in CS 7–8 channel, which was the primary atrial sensing channel. The program then evaluated the electrogram measured in CS 5–6 (secondary atrial sensing channel) and continued the ablation despite the noise. Without this cross check, the ablation would have stopped incorrectly because of “AV block”. This ablation run turned out to be successful with slow junctional rhythm observed subsequently.

**Figure 3:**
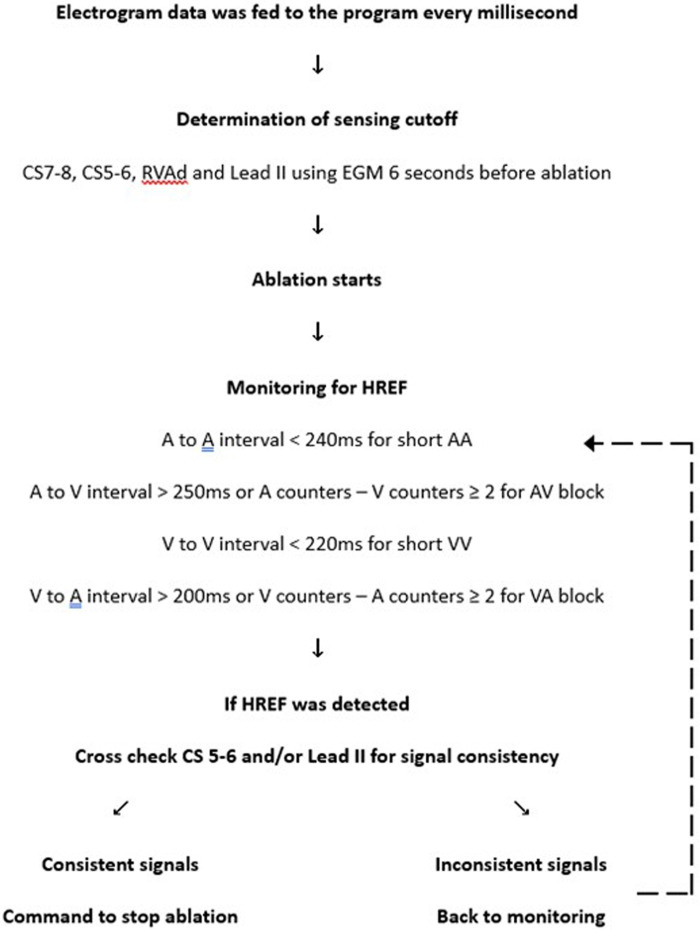
Schematics of the program.

Similar to pacemakers, the program had an auto-sensing adjustment feature to personalize the sensing of atrial and ventricular signals by analyzing 6 s of electrogram data before ablation started. The final atrial and ventricular sensing threshold was set at 50% of the smallest CS7–8 and RVAd electrograms respectively. Utilizing CS7–8 and RVAd channels as primary sensing sources, the atrial and ventricular counters were updated in real-time during the ablation run. There was a post-sensing blanking of 200 ms for both atrial and ventricular channels to avoid double counting of a broad electrogram. Although this led to 100% sensitivity, the specificity was only 86.8%. This reduced specificity was due to the catheters sometimes sensing noise during ablation and occasional premature beats may have diminished amplitudes. An example of such a case can be seen in Figure 2. These problems significantly reduced the specificity of the program. To tackle this issue, the CS5–6 and Lead II channels were used for supplementary sensing of atrial and ventricular signals respectively. When HREF was detected by the program, the CS5–6 and Lead II channels were cross-checked to ensure consistency of the signals before commanding ablation termination. As a result, the program ultimately used four out of the available 14 channels of data. A schematic rundown of the program is illustrated in Figure 3. After optimization, the program had a sensitivity of 92.3% and a specificity of 100% in the training set.

### Evaluation of program accuracy in validation set

**Figure 4:**
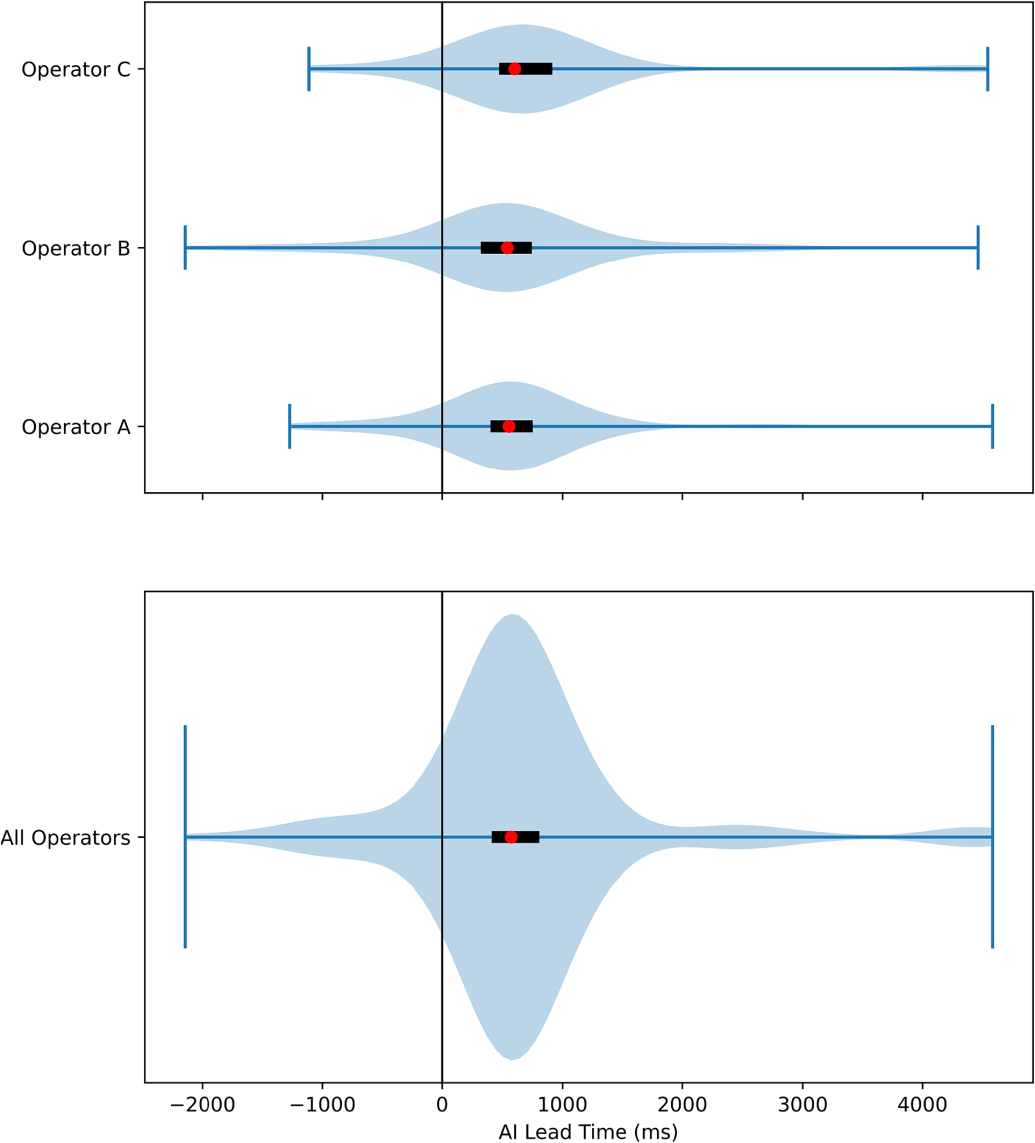
Violin plot of AI lead time. The distribution of AI Lead Time was plotted, with positive values favoring the AI. The red dots represented the median time, and the black rectangles were interquartile ranges. The result of each individual operator was plotted separately in the top plot, and the results from all three operators were combined in the bottom plot.

The validation set contained 206 ablation runs. One run was excluded due to incomplete recording before ablation (less than 6 s of EGM were recorded before ablation), and 4 runs were excluded due to significant noise in the RVAd channel before ablation started. Supplementary Figure S5 shows an example of an excluded run. Among the remaining 201 runs, three were further excluded from final analysis because consensus could not be reached by the three operators (Supplementary Figure S6). In the remaining 198 runs, 67 had HREF. A summary of the results from the validation set can be seen in Table 2. The mean sensitivity and specificity of the human operators were 93.5% and 95.5% respectively, which was comparable to that of the program at 95.2% and 91.1% respectively. In the runs where HREF were recognized by both human and program, ablations were terminated earlier by the program 90.2% of the times. The distribution of stop times for human operators and the program are shown in Figure 4. The median AI Lead Time was 574 ms (interquartile range 412–807 ms), and mean AI Lead Time was 673.7 ± 969.9 ms. These results demonstrate that the program was faster in recognizing HREF (*p* < 0.001 for both Wilcoxon Signed Rank Test and *t*-test).

**Table 2:** **Result of validation set**. *P*-values were calculated with Wilcoxon Signed Rank test and one sample *t*-test for median and mean respectively.

	Operator 1	Operator 2	Operator 3	Total human arm	AI
Sensitivity	0.970	0.984	0.851	0.935	0.952
Specificity	0.961	0.917	0.985	0.955	0.911
AI Lead Time					
Median (ms)	561	542	602	574	N/A
IQR (ms)	405.5	320.75	473	396	N/A
*P* value for median	<0.001	<0.001	<0.001	<0.001	N/A
Mean ± SD (ms)	613.9 ± 844.6	609.8 ± 1,012.9	824.1 ± 1,057.8	673.7 ± 969.9	N/A
*P* value for mean	<0.001	<0.001	<0.001	<0.001	N/A

Sensitivity analysis showed that if cutoff values were made 10% more lenient (275 ms for AV interval, 220 ms for VA interval, 216 ms for A-A interval and 198 ms for V-V interval) or 10% more stringent (225 ms for AV interval, 180 ms for VA interval, 264 ms for A-A interval and 242 ms for V-V interval), sensitivity and specificity of the program remained higher than 90%.

## Discussion

Our study utilized electrogram data gathered from ablation runs to design and validate a program that had comparable sensitivity and specificity to experienced electrophysiologists in recognizing high-risk electrogram features during slow pathway modification. The program was able to terminate ablation earlier than human intervention 90% of the time in response to HREF.

Permanent AV block, albeit uncommon, is the most dreadful complication that can occur during ablation around the AV node (7). While the occurrence of junctional beats is a sensitive feature to predict ablation success, fast junctional beats suggest fast pathway or compact AV node irritation. The presence of these should prompt immediate termination of the ablation (9). In earlier studies, the presence of VA block during ablation was also found to precede the development of subsequent AV block (10). They were all found to be common reasons for ablation termination in our training set.

High power ablations for as short as 2–4 s were able to create durable lesions in the atrium (15, 16). The AV node and conduction system, structures that should be avoided during ablation, are found at the atrial endocardial surface. This location makes them vulnerable to inadvertent damage during this procedure. Therefore, if HREF is present, terminating ablation milliseconds earlier can make an effective difference. This presents an opportunity where AI automation can be of assistance.

The program requires two proximal CS channels (CS7–8 and CS5–6) for atrial timing, and two channels (RVAd and lead II) for ventricular timing evaluation. The use of only four channels simplifies the program and minimizes processing time. This combination allows the program to stop ablation 90% of the time before human intervention. These four channels are also routinely recorded during slow pathway modification in most EP labs, thus making this program generalizable. In the future, the cutoff values can also be re-programmed to suit the needs of different patients.

### Limitations of the study

**Figure 5:**
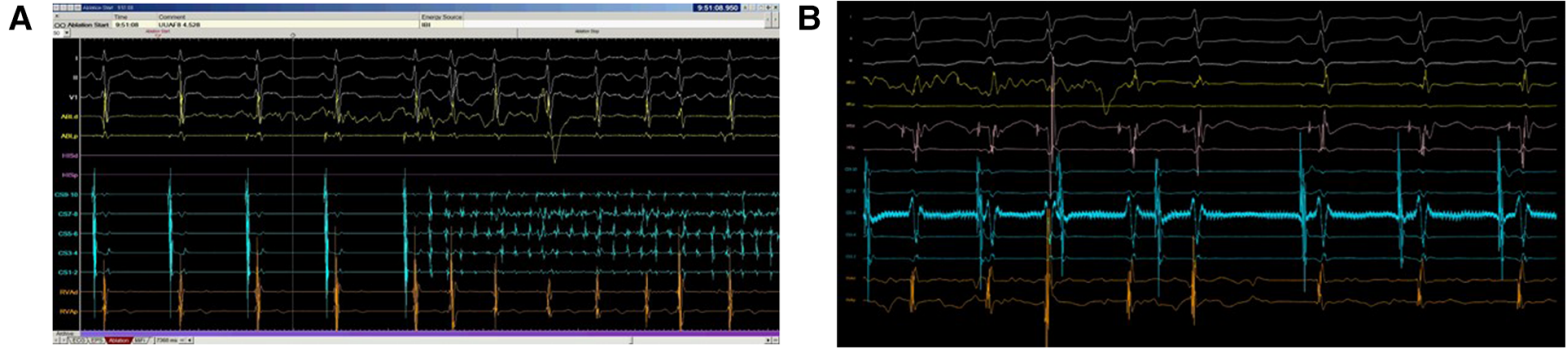
Program failed to detect HREF. In the top panel **(A)**, ablation should be stopped in response to atrial fibrillation as AV conduction could not be safely monitored. However, as the signal amplitude in both CS 7–8 and CS 5–6 were small, the program was unable to sense atrial fibrillation properly. The bottom panel **(B)** showed HREF with VA block but far-field electrogram with large amplitude in CS 5–6 confused the program sensing. Both scenarios caused significant delay in ablation termination.

First and foremost, although the program can achieve comparable accuracy to an experienced electrophysiologist, it is not perfectly accurate because of unexpected changes in electrogram amplitude and noise during ablation. For example, the program may undersense atrial fibrillation resulting in a delay to terminate ablation. Large far field electrogram may also confuse electrogram sensing (Figure 5). Therefore, real-time monitoring by the electrophysiologist is still irreplaceable. The goal of the program was not to replace the judgement of an experienced electrophysiologist. This innovation is analogous to developing a self-driving car. While the car can turn and brake automatically before a crash, the human driver is ultimately responsible to still have their hands on the steering wheel. It is expected that human intervention will not be frequently required as sensitivity is 95.2% and the program can terminate the ablation earlier than a human 90.2% of the time. Inadvertent premature termination of a successful ablation run occurred in 8.9% of the validation set. In clinical practice, premature ablation termination can be restarted in due course, resulting in no additional harm apart from a few seconds of procedure time prolongation.

Secondly, this study is a single center retrospective study. The three electrophysiologists testing the validation set videos and adjudicating HREFs were blinded from the program design to combat the inherent limitation of a retrospective study design. Although the cutoff values were generated from the training set obtained from a single center, sensitivity analysis demonstrated that variation of these values by 10% still offered acceptable sensitivity and specificity of the program (>90%). To avoid selection bias, consecutive ablation procedures were included and the exclusion criteria were limited. Only 5 out of 206 runs were excluded from analysis in the validation set. These excluded runs all had unacceptable electrogram qualities prior to ablation starting. In a real-world setting, the operator would need to reposition the catheters and check all connections to ensure clear electrograms can be obtained, allowing for optimized input into the program. As all the electrograms were generated by one recording system, the program would require slight modifications before application to another EP recording system.

Thirdly, the results generated in this study would not be directly applicable for ablation in a pediatric population as they may have different cutoff values for AV conduction. Cryoablation procedures may not produce junctional rhythms and therefore also cannot be monitored by this program.

**Figure 6:**
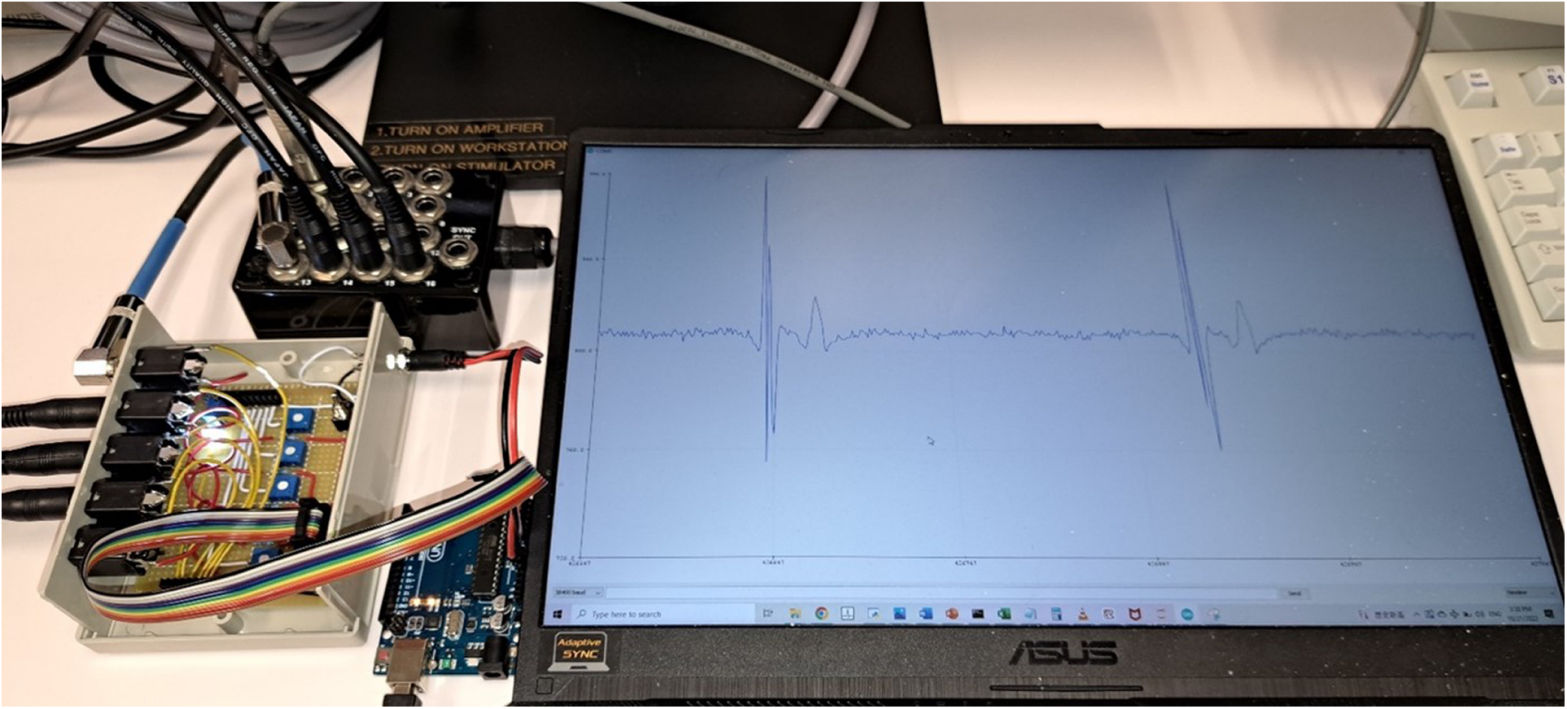
Prototype of the AI program for prospective testing. The four analog output channels were connected to a circuit board for signal amplification and rectification. The whole unit was then connected to a microcontroller board (Arduino Uno Rev 3). The analog signal was converted to digital signal by the microcontroller and fed into the computer program. The computer screen was displaying one of the four input channels in real-time (CS7–8 in this case).

With the promising results generated from this retrospective study, prospective evaluation on human subjects is the next step. For this, a prototype has been developed using a simple microcontroller board (Arduino Uno Rev 3, Monza, Italy) with built-in Analogue to Digital Converter (ADC) of 10-bits resolution, as shown in Figure 6. The analogue output of the EP recording system will be connected to a signal amplifier. The amplified signal will then be connected to electrode pins of the microcontroller and go through the ADC. The digital signal can then be transmitted to the computer and fed into the program. If HREF is recognized, a signal can be sent to the ablation pedal to open the circuit and terminate the ablation. This prototype can potentially be used in all EP recording systems that have at least 4 analogue output channels.

## Conclusion

A program was developed to monitor HREF with similar accuracy to a human operator. This program was able to terminate ablation earlier than human intervention 90% of the time in response to HREF with a median lead time of 574 ms. A prospective study is required to test this program in clinical use.

## Data Availability

The raw data supporting the conclusions of this article will be made available by the authors, without undue reservation.

## Supplementary material

Supplementary Figure S1 Ablation termination by program due to short AA time of 228 ms. Termination occurs when AA time is less than the program cutoff value of 240 ms.

Supplementary Figure S2 Ablation termination by program due to short VV time of 209 ms. Termination occurs when VV time is less than the program cutoff value of 220 ms.

Supplementary Figure S3 Ablation termination by program due to AV block. Termination occurs when AV interval exceeds the cutoff value of 250 ms.

Supplementary Figure S4 Ablation termination by program due to VA block. Termination occurs when VA interval exceeds the cutoff value of 200 ms.

Supplementary Figure S5 Example of an excluded run. Four out of five excluded runs in the validation set were excluded from validation set analysis due to significant noise in the RVAd channel before start of ablation. These four runs all belonged to the same patient, an example of which is shown here. One remaining run was excluded because the record was incomplete with less than 6 seconds of pre-ablation electrogram being recorded.

Supplementary Figure S6 Example of an excluded run in the validation set. Two operators decided ablation needed to be stopped in response to fast premature atrial complexes, while one operator allowed it to continue. Consensus was not reached. The program did not regard it as HREF.

Supplementary Video S1 Demonstration of video simulation and calculation of AI Lead Time. 201 ablation runs in the validation set were rendered into ablation simulation videos at a frame rate of 100 Hz and played to three individual electrophysiologists. A foot pedal was used to control the video. Stepping down on the pedal would begin playing the video and releasing the pedal would stop the video. Upon pedal release, the time at the left lower corner would be recorded as the ablation stop time. This was compared with the ablation stop time determined by the program to calculate the AI Lead Time. A sample of the ablation simulation video can be found in supplementary material.
