# Supplementary figures and images for: Automatic algorithmic driven monitoring of atrioventricular nodal re-entrant tachycardia ablation to improve procedural safety
Source: Front Cardiovasc Med. 2023 Jul 3;10:1212837. doi: 10.3389/fcvm.2023.1212837 (PMC10352454; doi:10.3389/fcvm.2023.1212837)

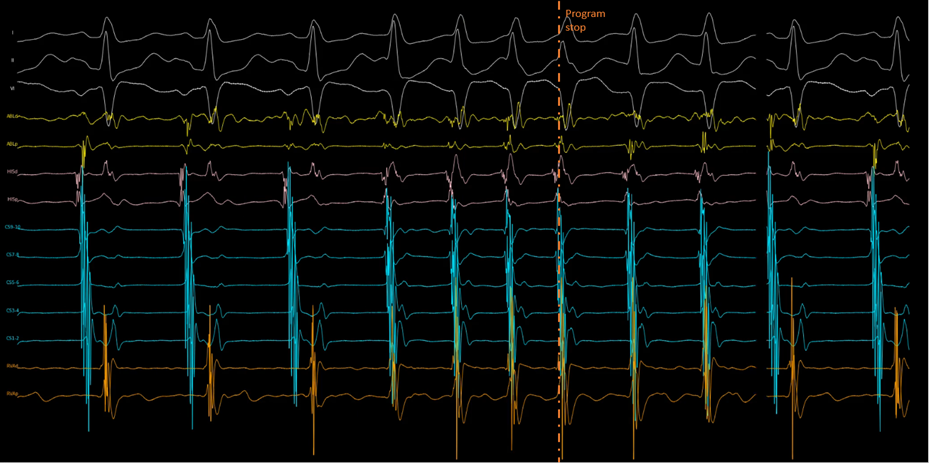

Supplement: Supplementary Figure S1 — Ablation termination by program due to short AA time of 228 ms. Termination occurs when AA time is less than the program cutoff value of 240 ms. [file Image1.png]

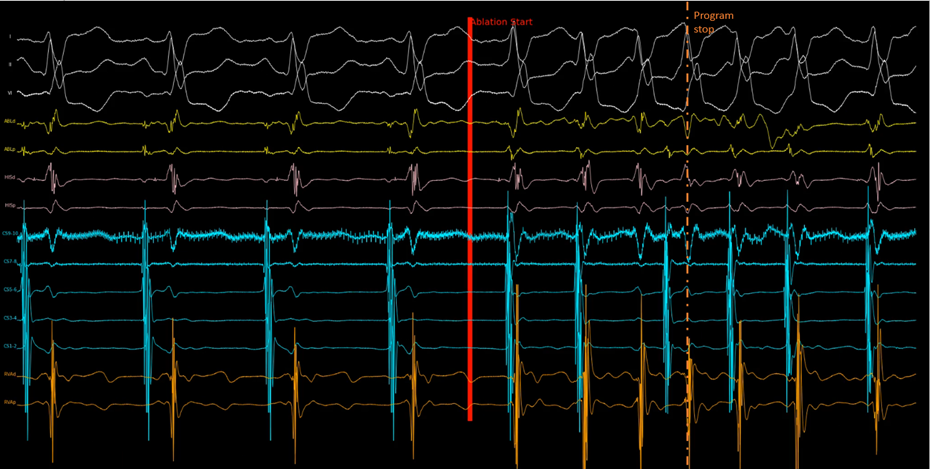

Supplement: Supplementary Figure S2 — Ablation termination by program due to short VV time of 209 ms. Termination occurs when VV time is less than the program cutoff value of 220 ms. [file Image2.png]

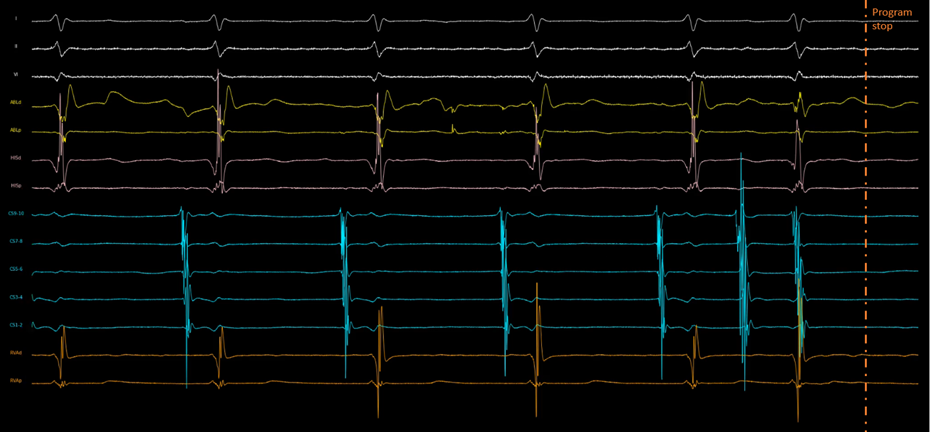

Supplement: Supplementary Figure S3 — Ablation termination by program due to AV block. Termination occurs when AV interval exceeds the cutoff value of 250 ms. [file Image3.png]

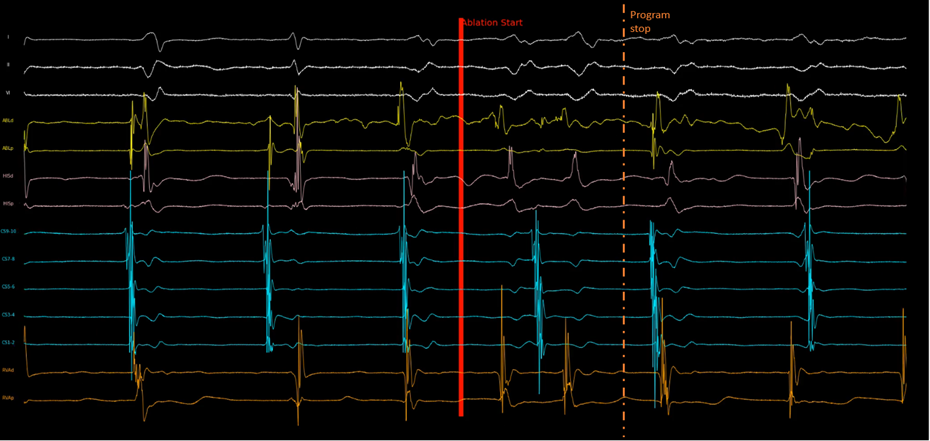

Supplement: Supplementary Figure S4 — Ablation termination by program due to VA block. Termination occurs when VA interval exceeds the cutoff value of 200 ms. [file Image4.png]

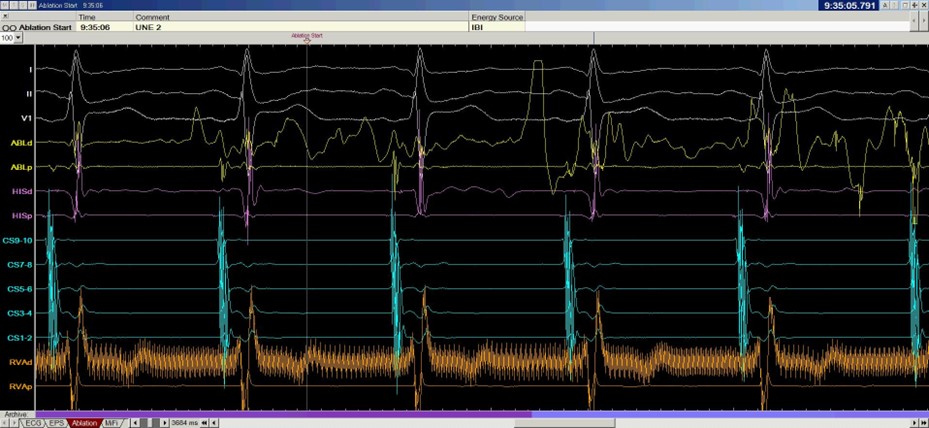

Supplement: Supplementary Figure S5 — Example of an excluded run. Four out of five excluded runs in the validation set were excluded from validation set analysis due to significant noise in the RVAd channel before start of ablation. These four runs all belonged to the same patient, an example of which is shown here. One remaining run was excluded because the record was incomplete with less than 6 seconds of pre-ablation electrogram being recorded. [file Image5.jpg]

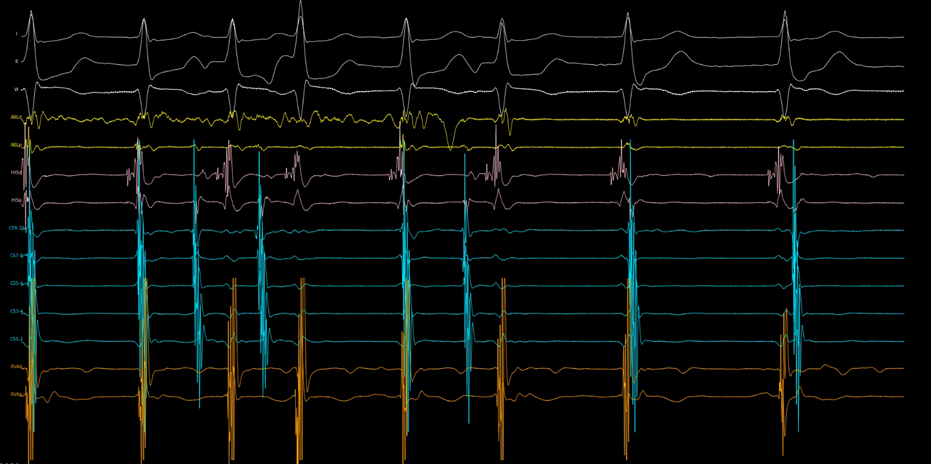

Supplement: Supplementary Figure S6 — Example of an excluded run in the validation set. Two operators decided ablation needed to be stopped in response to fast premature atrial complexes, while one operator allowed it to continue. Consensus was not reached. The program did not regard it as HREF. [file Image6.png]
